# Are Plasma Thyroid-Stimulating Hormone Levels Associated with Degree of Obesity and Metabolic Syndrome in Euthyroid Obese Patients? A Turkish Cohort Study

**DOI:** 10.1155/2014/803028

**Published:** 2014-01-02

**Authors:** Okan Bakiner, Emre Bozkirli, Gulhan Cavlak, Kursad Ozsahin, Eda Ertorer

**Affiliations:** ^1^Department of Endocrinology and Metabolism Diseases, Faculty of Medicine, Baskent University, Dadaloglu Mah Serinevler, 01250 Adana, Turkey; ^2^Department of Family Physician, Faculty of Medicine, Baskent University, Dadaloglu Mah Serinevler, 01250 Adana, Turkey

## Abstract

We aimed to observe the association between degree of obesity and metabolic syndrome and plasma thyrotropin levels in obese, euthyroid Turkish patients. 947 obese and overweight patients who admitted to our outpatient clinic were assessed retrospectively. 150 healthy euthyroid cases were also recruited as the control group. Cases with metabolic syndrome were determined. Patients were divided into various subgroups as overweight, obese, morbid obese, men, and women. No statistical significance was determined when all the patients' and subgroups' plasma thyrotropin levels were compared to normal weight control group. No association was shown between the presence of metabolic syndrome and plasma thyrotropin levels for both all patients and subgroups. Also there was not any association between each component of metabolic syndrome and plasma thyrotropin levels. In conclusion, we did not found any significant association between plasma thyrotropin levels and obesity and metabolic syndrome in our euthyroid subjects.

## 1. Introduction

Obesity is a serious health problem closely associated with insulin resistance and metabolic syndrome [1]. It has a complex pathophysiology characterized by interaction of genetic, environmental, and physiological factors [2]. Thyroid dysfunction is very well known to contribute to the pathogenesis of obesity. Weight loss is a common manifestation of hyperthyroidism and its treatment results in weight gain about four kilograms per year [3]. In contrast, weight gain is frequently seen in hypothyroidism and treatment causes mild weight loss [4]. The clinical observations mentioned above raise the questions of whether thyroid-stimulating hormone (TSH) change in physiological limits is associated with obesity and whether there is a link between adipose tissue and hypothalamo-thyroidal axis [5, 6]. Some studies have shown positive correlation between obesity and plasma TSH levels in euthyroid subjects; nevertheless, conflicting studies are also present [7–10].

Metabolic syndrome is an important predictor of cardiovascular risk and is a cluster of abnormalities such as abdominal obesity, dyslipidemia, high plasma glucose, and hypertension [11]. Besides their impact on energy homeostasis, thyroid hormones are known to have a variety of effects on metabolic syndrome determinants [12–14]. There are some studies which show an association between thyroid hormone levels and metabolic syndrome in euthyroid patients, but the presence of counter studies creates discrepancy [15, 16]. Conflicting results of different studies may be attributed to many contributing factors, such as ethnic differences, [17–23].

Herein, we aimed to determine the possible association among obesity, metabolic syndrome, and plasma TSH levels in our overweight or obese euthyroid cohort.

## 2. Materials and Methods

### 2.1. Patients and Methods

The participants of this retrospective study were the cases admitted to our outpatient clinic between January 2009 and April 2011 for obesity. Exhibiting a Body mass index (BMI) ≥ 25 kg/m^2^ and plasma TSH levels between 0.3–5 mIU/mL were sought for inclusion. Cases with known thyroidal disease, history of thyroidectomy, accompanying endocrinological pathology, and/or history of receiving antithyroid medication, levothyroxine, or drugs which interact with thyroid functions were excluded. A group of 150 healthy euthyroid age- and gender-matched cases (31 males, 119 females) with BMI below 25 was included as control group.

Antropometric and blood pressure measurements, plasma TSH, simultaneous fasting plasma glucose, triglycerides, and high density lipoprotein cholesterol (HDL) levels of the patients were supplied from hospital records. For the control group, blood samples were taken early in the morning from antecubital vein after 12 hours of fasting. Arithmetical mean of three consecutive blood pressure measurements following five minutes of rest was recorded. Waist circumference was obtained from both suprailiac crests. Fastening body weight and height were measured and recorded. BMI values were calculated as kg/m^2^. Body fat percentages of the patients were obtained early in the morning with TANITA BF300 foot to foot bicompartmental bioelectrical impedance technique after an overnight fasting following miction.

Plasma glucose was assayed using the enzymatic glucose oxidase method (Roche Modular, Roche Diagnostics GmbH, Mannheim, Germany) with intra- and interassay coefficients of variation (CVs) 1.0 and 1.7%, respectively. The HDL cholesterol and triglycerides concentrations were determined enzymatically by cholesterol oxidase-peroxidase and glycerol phosphate dehydrogenase-peroxidase methods, respectively with the same autoanalyser and CVs less than 5%.

Serum TSH levels were measured by chemiluminescence assay method using an Abbott-Architect analyser (Chicago, IL, USA). The CVs for intra- and interassay imprecision were 3% and 6%, respectively.

The diagnosis of metabolic syndrome was performed using the National Cholesterol Education Program-Adult Treatment Panel III criteria [24].

### 2.2. Study Design

Patients were separated into subgroups according to BMI and gender as shown in Table 1. Healthy volunteers participating in the study (*n* = 150) were accepted as the control group. Plasma TSH levels of the control group were compared with all patients and subgroups. The association between plasma TSH levels and BMI, and waist circumference, and body fat percentages were investigated in all groups. The same procedure was performed for TSH levels and metabolic syndrome and its determinants, as well. Then, the TSH level 2.5 mIU/mL was accepted as a cut-off according to Wartofsky's data, the ones with TSH over this limit were accepted as high normal TSH group, whereas, the cases below this value as low normal TSH group [25]. The relationship between TSH and obesity and presence of metabolic syndrome was investigated between these two new TSH groups.

### 2.3. Ethics

Baskent University ethics committee which conforms to the provisions of the Declaration of Helsinki in 1995 (as revised in Edinburgh 2000) approved the study protocol with the tracking number KA 12/279. Written informed consent was obtained from each healthy participant and patient anonymity was preserved for all subjects.

### 2.4. Statistical Analysis

Statistical analysis was performed using the statistical package SPSS v 17.0. For each continuous variable, normality was checked by the Kolmogorov-Smirnov and Shapiro-Wilk tests and by histograms. Comparisons between groups were applied using Student's *t*-test for normally distributed data and Mann-Whitney *U* test was used for the data not normally distributed. The categorical variables between the groups were analyzed using the Chi-square test or Fisher Exc. test. A *P* value of 0.05 was taken as the level of significance.

## 3. Results

A total of 947 patients were studied retrospectively and 629 patients were found to be eligible for inclusion. Demographic characteristics of the study and control groups were given in Table 2.

There were positive correlations between waist circumference and BMI, BMI and body fat percentage, and waist circumference and body fat percentage (*r* = 0.76, *r* = 0.54, *r* = 0.31, for all *P* = 0.001, resp.). There was a significant correlation between the presence of metabolic syndrome and waist circumference, BMI, and body fat percentage for all patients and all of the subgroups (*P* = 0.001).

No statistical significant difference was determined when group 1, group 2, and group 3 as well as whole patient group plasma TSH levels were compared to normal weight control group (*P* = 0.34, *P* = 0.69, *P* = 0.12, and *P* = 0.47, resp.). Comparison of plasma TSH levels in subgroups was given in Table 3. There was no significant difference in subgroups according to plasma TSH levels.

Plasma TSH levels and anthropometric parameters did not exhibit any significant correlation in all patients and subgroups (Table 4).

No association was shown between the presence of metabolic syndrome and plasma TSH levels (Table 4). When association between each component of metabolic syndrome and plasma TSH levels was studied, no correlation was found (Table 5).

The data of high normal TSH group and low normal TSH group is given in Table 4. No significant difference was found between anthropometric parameters of these new groups (Table 6). Similar number of patients in each group had metabolic syndrome (*P* = 0.37).

## 4. Discussion

In our euthyroid cohort, overweight, obese, morbid obese, and normal weight subjects did not demonstrate any association regarding BMI categories and plasma TSH levels. No association was detected between plasma TSH levels and the presence of metabolic syndrome, as well. This finding applied for both sexes. Cases with high normal and low normal TSH levels exhibited no difference in terms of obesity categories and metabolic syndrome.

Although the significant association between overt hypothyroidism and high adiposity has been demonstrated before, the issue is not clear for cases with TSH levels within normal limits [26]. A meta-analysis by Souza and Schieri showed that 18 studies out of 29 on this topic have demonstrated a significant association between plasma TSH levels and adiposity, but those were not supported by the others [27]. In this meta-analysis, the correlation between TSH and obesity has been found in 11 of the clinical studies and it has been valid for both sexes in presence of morbid obesity and only for females in presence of obesity. As there are no different gender-specific cut-off values for normal plasma TSH, the correlation found only for obese women but not for men cannot be explained. Likewise, we could not find any correlation between BMI and plasma TSH levels for each gender.

Scientists supporting the idea that body adiposity correlates with increasing plasma TSH levels in euthyroid subjects have proposed that upper limit of normal TSH levels may be presumed as 2.5 mIU/mL and upper values may resemble subclinical hypothyroidism [25]. However in a wide population-based study conducted by Hamilton and co-workers, the upper limit of plasma TSH has been proposed as 4.0 mIU/mL for those with no thyroidal disease findings [28]. In accordance with this study, we did not determine any difference in terms of body adiposity when a TSH cut-off level 2.5 mIU/mL was used.

Some studies have pointed out the elevation of metabolic syndrome risk in euthyroid cases with the increasing TSH levels [29, 30]. However, another study has shown no increase of metabolic syndrome incidence even in subclinical hypothyroidism [31]. In the present study, there was no correlation between plasma TSH levels and the presence of metabolic syndrome and each component of metabolic syndrome. Different results of different studies including ours may be related to distributional differences of groups, sensitivity of TSH measurement techniques, changes in reference cut-off points and race differences of normal serum TSH levels with definition of obesity, and metabolic syndrome [18–23].

Our study does not include free thyroid hormone levels, thyroid autoantibody levels, and lack of reevaluation for patients who lost weight after life style interventions which can be regarded as the pitfalls of the study. However plasma TSH assay seems to be the most useful laboratory test in the initial evaluation of thyroid disfunction in monitoring patients for thyroid disease. In addition, free thyroid hormone levels and autoantibody measurements are secondary diagnostic steps and are not mandatory for screening [32]. Our patients were admitted to the outpatient clinic for obesity and other parameters except TSH were not evaluated. Furthermore, this study was planned via retrospective data and follow-up findings are not included.

Absence of correlation between plasma TSH levels and obesity in the present study does not mean that TSH and thyroid hormones have no effect on adiposity. Recent studies have shown extrathyroidal TSH receptors on adipose tissue [33, 34]. The TSH receptors found on rat preadipocytes have been demonstrated to play a role on adipocyte differentiation. Increasing TSH receptor expression in human subcutan adipose tissue has been shown to be parallel to the severity of obesity [33, 35]. These findings suggest that TSH-adiposity interaction may be related to local TSH receptor expression rather than fluctuating plasma TSH levels. Dentice and coworkers have defined the importance of type II deiodinase activity for myogenesis and muscle regeneration [36]. Boelen and colleagues have reported that local deiodinase activity can change as an adaptation for differing situations and this may regulate the local effects of thyroid hormones [37]. Additionally, differences in local expressions of thyroid hormone receptors may be related to adipogenesis similar to local TSH receptor expression. Accordingly, it has been demonstrated that obese cases had a significant raise in thyroid hormone receptor alpha-1 gene expression in subcutaneous fat tissue compared to omental fat tissue [38].

Taking our results and the available medical literature into consideration, we think that the association between plasma TSH levels and obesity and metabolic syndrome may not be related to plasma hormone levels but probably to local hormone efficiency. Studies on local hormone effects in euthyroid obese models will clarify this issue.

## Figures and Tables

**Table 1:** Subgroups according to BMI and gender.

Group	Name of group	BMI (kg/m^2^)	*n*
1	Overweight	25–29.9	133
2	Obese	30–39.9	330
3	Morbid obese	≥40	166
4	Men only	≥25	102
5	Women only	≥25	527

**Table 2:** Demographic data of all patients, subgroups, and control group. The data were reported as mean ± StdDev.

Characteristics	All patients	Group 1	Group 2	Group 3	Group 4	Group 5	Controls
Number of patients (*n*)	629	133	330	166	102	527	150
Age (years)	37.13 ± 11.18	34.04 ± 9.83	36.68 ± 10.55	40.92 ± 12.3	36.06 ± 10.9	37.33 ± 11.2	36.67 ± 10.33
Weight (kg)	94.99 ± 20.16	74.73 ± 7.50	92.44 ± 12.32	117.28 ± 17.57	114.21 ± 23.13	91.27 ± 17.23	67 ± 12.41
Waist circumference (cm)	108.87 ± 14.65	96.86 ± 7	106.78 ± 10.18	123.52 ± 14.25	121.27 ± 16.81	106.47 ± 12.89	87 ± 8.42
BMI (kg/m^2^)	36.01 ± 7.15	27.92 ± 1.3	34.73 ± 2.79	45.48 ± 4.81	37.45 ± 6.95	35.74 ± 7.16	23.18 ± 1.49
TANITA body-fat percentage (%)	41.24 ± 8.12	35.01 ± 4.57	40.72 ± 5.92	47.10 ± 5.88	35.13 ± 7.72	42.42 ± 7.66	24.89 ± 7.43
TSH (mIU/mL)	1.83 ± 0.94	1.63 ± 0.77	1.84 ± 0.94	1.94 ± 1.02	1.71 ± 0.77	1.85 ± 0.97	1.69 ± 0.89
Patients with metabolic syndrome (*n*)	330	35	179	116	58	272	0
Patients with hypertension (*n*)	119	5	51	63	24	95	0
Fasting plasma glucose (mg/dL)	97.36 ± 13.65	93.16 ± 7.7	97.12 ± 12.92	101.74 ± 17.12	100.85 ± 15.32	96.68 ± 13.21	89 ± 6.9
Triglyceride (mg/dL)	145.46 ± 83.47	124.18 ± 78.04	147.68 ± 80.32	163.71 ± 90.02	183.68 ± 101.11	138.06 ± 77.58	121.12 ± 42.87
HDL cholesterol (mg/dL)	45.93 ± 12.04	49.33 ± 11.48	45.49 ± 11.47	42.86 ± 11.3	39.62 ± 11.1	47.16 ± 11.84	50.02 ± 14.21

**Table 3:** Comparison of plasma TSH levels in subgroups. Results were given as *P* value in each column. A *P* value of 0.05 was taken as the level of significance.

	Group 1	Group 2	Group 3	Group 4	Group 5	Control
Group 1	—	0.39	0.29	0.07	0.09	0.34
Group 2	0.39	—	0.43	0.72	0.41	0.69
Group 3	0.29	0.43	—	0.67	0.49	0.12
Group 4	0.07	0.72	0.67	—	0.21	0.29
Group 5	0.09	0.41	0.49	0.21	—	0.36

Control	0.34	0.69	0.12	0.29	0.36	—

**Table 4:** The association between anthropometric measures and plasma TSH levels for total-patient group and subgroups.

Groups	BMI-TSH	Weight-TSH	TANITA body fat percentage-TSH	Waist circumference-TSH
	(kg/m^2^-mIU/mL)	(kg-mIU/mL)	(%-mIU/mL)	(cm-mIU/mL)
All patients	*r* = 0.054	*r* = 0.037	*r* = 0.023	*r* = 0.046
	*P* = 0.28	*P* = 0.16	*P* = 0.81	*P* = 0.74
Group 1	*r* = − 0.15	*r* = − 0.154	*r* = − 0.123	*r* = − 0.054
	*P* = 0.08	*P* = 0.22	*P* = 0.19	*P* = 0.064
Group 2	*r* = −0.043	*r* = − 0.70	*r* = − 0.077	*r* = − 0.027
	*P* = 0.43	*P* = 0.27	*P* = 0.09	*P* = 0.73
Group 3	*r* = − 0.39	*r* = − 0.417	*r* = − 0.005	*r* = 0.012
	*P* = 0.36	*P* = 0.47	*P* = 0.81	*P* = 0.02
Group 4	*r* = 0.267	*r* = 0.235	*r* = 0.117	*r* = 0.17
	*P* = 0.007	*P* = 0.017	*P* = 0.36	*P* = 0.09
Group 5	*r* = 0.027	*r* = 0.031	*r* = 0.01	*r* = 0.051
	*P* = 0.077	*P* = 0.62	*P* = 0.56	*P* = 0.12

**Table 5:** The relationship between plasma TSH levels and the presence of metabolic syndrome and its components in all patients and subgroups.

Groups	TSH (mIU/mL)-metabolic syndrome	TSH (mIU/mL)-fasting plasma glucose (mg/dL)	TSH (mIU/mL)- hypertension	TSH (mIU/mL)-triglyceride (mg/dL)	TSH (mIU/mL)-HDL cholesterol (mg/dL)
All patients	*P* = 0.47	*r* = 0.057 *P* = 0.24	*P* = 0.49	*r* = 0.052 *P* = 0.33	*r* = −0.101 *P* = 0.011
Group 1	*P* = 0.36	*r* = 0.093 *P* = 0.61	*P* = 0.36	*r* = 0.041 *P* = 0.08	*r* = −0.137 *P* = 0.86
Group 2	*P* = 0.63	*r* = 0.069 *P* = 0.06	*P* = 0.17	*r* = 0.021 *P* = 0.052	*r* = −0.053 *P* = 0.49
Group 3	*P* = 0.09	*r* = −0.052 *P* = 0.054	*P* = 0.51	*r* = 0.113 *P* = 0.91	*r* = −0.144 *P* = 0.56
Group 4	*P* = 0.16	*r* = 0.074 *P* = 0.98	*P* = 0.92	*r* = 0.059 *P* = 0.048	*r* = −0.23 *P* = 0.018
Group 5	*P* = 0.27	*r* = 0.6 *P* = 0.23	*P* = 0.63	*r* = 0.051 *P* = 0.17	*r* = −0.85 *P* = 0.49

**Table 6:** The data of the group with plasma TSH level >2,5 mIU/mL (high normal TSH group) and of the group with plasma TSH level <2,5 mIU/mL (low normal TSH group). The data were reported as mean ± Standard deviation.

Parameters	High normal TSH group	Low normal TSH group	*P*
Number of cases (*n*)	142	487	0.002
TSH (mIU/mL)	3.25 ± 0.61	1.42 ± 0.54	0.003
BMI (kg/m^2^)	36.9 ± 6.6	35.7 ± 7.2	0.87
Body weight (kg)	95.9 ± 19.6	94.7 ± 20.3	0.12
Waist circumference (cm)	109.3 ± 13.7	108.7 ± 14.9	0.65
Body fat percentage calculated with TANITA (%)	42.7 ± 9.6	40.9 ± 7.5	0.076
Cases with metabolic syndrome (*n*)	84	246	0.24
Fasting plasma glucose (mg/dL)	99.1 ± 15.3	96.3 ± 13.0	0.63
Triglyceride (mg/dL)	156.3 ± 99.5	142.2 ± 77.9	0.31
HDL cholesterol (mg/dL)	44.2 ± 10.3	46.4 ± 12.4	0.19
Cases with hypertension (*n*)	25	94	0.061

## Abbreviations

TSH: Thyroid-stimulating hormone
CVs: Coefficients of variation
HDL: High-density lipoprotein cholesterol
BMI: Body mass index.
